# Insight Into the Variation of Bacterial Structure in Atrazine-Contaminated Soil Regulating by Potential Phytoremediator: *Pennisetum americanum* (L.) K. Schum

**DOI:** 10.3389/fmicb.2018.00864

**Published:** 2018-05-04

**Authors:** Bo Cao, Ying Zhang, Ziyi Wang, Mengyuan Li, Feng Yang, Duo Jiang, Zhao Jiang

**Affiliations:** School of Resources and Environment, Northeast Agricultural University, Harbin, China

**Keywords:** rhizosphere, atrazine, Pennisetum, phytoremediation, microbial succession

## Abstract

Although plants of the genus *Pennisetum* can accelerate the removal of atrazine from its rhizosphere, the roles played by this plant in adjusting the soil environment and soil microorganism properties that might contribute to pollutant removal are incompletely understood. We selected *Pennisetum americanum* (L.) K. Schum (*P. americanum*) as the test plant and investigated the interaction between *P. americanum* and atrazine-contaminated soil, focusing on the adjustment of the soil biochemical properties as well as bacterial functional and community diversity in the rhizosphere using Biolog EcoPlates and high-throughput sequencing of the 16S rRNA gene. The results demonstrate that the rhizosphere soil of *P. americanum* exhibited higher catalase activity, urease activity and water soluble organic carbon (WSOC) content, as well as a suitable pH for microorganisms after a 28-day incubation. The bacterial functional diversity indices (Shannon and McIntosh) for rhizosphere soil were 3.17 ± 0.04 and 6.43 ± 0.86 respectively, while these indices for non-rhizosphere soil were 2.95 ± 0.06 and 3.98 ± 0.27. Thus, bacteria in the *P. americanum* rhizosphere exhibited better carbon substrate utilization than non-rhizosphere bacteria. Though atrazine decreased the richness of the soil bacterial community, rhizosphere soil had higher bacterial community traits. For example, the Shannon diversity indices for rhizosphere and non-rhizosphere soil were 5.821 and 5.670 respectively. Meanwhile, some bacteria, such as those of the genera *Paenibacillus*, *Rhizobium*, *Sphingobium*, and *Mycoplana*, which facilitate soil nutrient cycling or organic pollutants degradation, were only found in rhizosphere soil after a 28-day remediation. Moreover, redundancy analysis suggests that the soil biochemical properties that were adjusted by the test plant exhibited correlations with the bacterial community composition and functional diversity. These results suggest that the soil environment and bacterial properties could be adjusted by *P. americanum* during phytoremediation of atrazine-contaminated soil.

## Introduction

Atrazine (2-chloro-4-ethylamino-6-isopropylamino-1,3,5-triazine) is one of the most widely used herbicides in agriculture. It is primarily applied to control broadleaf weeds in the crops such as maize, sorghum and sugar cane. Though atrazine has been proved highly persistent in the environment with the reported half-life ranges between 10 and 5824 days (Salazar-Ledesma et al., 2018), it also could be metabolized in environment according to microbiological degradation and some kinds of physicochemical process (Sun et al., 2010; Roustan et al., 2014). As a result, atrazine and its metabolites are the most commonly detected pesticide contaminants in groundwater and surface water due to their mobility in soil (Yang et al., 2014). In addition, atrazine has been classified as a priority pollutant since many researches proved it could affect the endocrine system of various kinds of organisms (Lasserre et al., 2009). In addition, there were also some other reports reveal that atrazine also could cause obvious toxic affection on the microorganisms in soil (Muñoz-Leoz et al., 2011; Imfeld and Vuilleumier, 2012). As a result, the soil nutrients cycling, as well as soil physical and chemical properties were also affected simultaneously. Consequently, the toxicity of atrazine has raised serious concerns and innovative strategies for remediating atrazine-contaminated soils are critically needed (Lima et al., 2009; Pandey et al., 2009).

In recent years, phytoremediation has aroused increasing concern in the field of contaminated soil remediation (Hamdi et al., 2012; Wang et al., 2012; Albright et al., 2013; Jagtap et al., 2014). Unlike the conventional physical or chemical technologies for soil remediation, which have the disadvantages of high economic costs, formulation of secondary contaminants and damage to soil organisms, phytoremediation has been considered a cost-effective, environmentally friendly strategy to solve soil contamination (Agnello et al., 2016; Gil-Díaz et al., 2016). Several literatures have been reported that phytoremediation can decrease the residual level of organic contaminates in soil by the interaction between plant roots and the specific microorganisms harbored in the rhizosphere (Cai et al., 2010). The plant-stimulated bioremediation of organic pollutants by rhizospheric microorganisms described above is also termed rhizoremediation (Hussain et al., 2018). Rhizoremediation is the major mechanism for phytoremediation of organic-polluted soil because it stimulates the population growth and activity of degrading microorganisms around roots through the rhizosphere effect (Truu et al., 2015). It is well known that the plant roots can create a nutrient-rich micro-environment for pollutants-degrading microbes, as well as that the microorganisms in rhizosphere can enhance plant growth by providing plant nutrients and protection against the stress caused by contaminants (Hussain et al., 2018; Lacalle et al., 2018). Therefore, the rhizosphere has long been considered as the most biologically active microsites in soil and the organic compounds are degraded here by the stimulated microbial biomass and the activity that is part of the rhizosphere effect (Liu et al., 2014).

Root exudates, which consist of low-molecular-weight carbohydrates, amino acids and organic polymers, can be used as an energy and carbon source by soil microbes during their metabolic processes (Martin et al., 2014). Therefore, root exudation is considered as a potential driving force for stimulated rhizoremediation and is the most important factor affecting microbial drift in the rhizosphere (Hussain et al., 2018). The distinct microorganism community shifts in contaminated soils mentioned above suggest the alteration of microbial catabolic activity and selection of specific microbial strains. Therefore, it is widely considered that root exudates play a distinctive role in shaping the rhizosphere microbiome in polluted soil (Thijs et al., 2016).

The selection of a suitable plant species is an indispensable component of phytoremediation success (Lacalle et al., 2018). It is widely supposed that a plant with the potential for organic pollution remediation may selectively enrich the specific pollutant degraders harbored in rhizosphere by releasing a variety of root exudates (Fang et al., 2001). In addition, the different plant select for certain microorganism or sharpen characteristics, and the types of microorganism that thrive on the rhizoplane mainly depend on the plant types (Chen et al., 2016). This is mainly because the constituents or the concentrations of the root exudates might different between the various species of plants (El Amrani et al., 2015). Therefore, further investigation of the distinct microorganism community or activity stimulating traits by the selected plant used for rhizoremediation is essential to illustrate the phytoremediation mechanism of contaminated soil.

The genus *Pennisetum* has been useful for remediation of soils contaminated with atrazine (Singh et al., 2004). However, little information about the interaction between the *Pennisetum* genus plant roots and the soil microorganisms, especially how this plant affects the soil biochemical properties or bacterial functional diversity and community structure, is available. The objectives of this study were (1) to assess the impact of the *Pennisetum americanum* (L.) K. Schum (*P. americanum*) rhizosphere on atrazine-contaminated soil biochemical properties, and (2) to investigate the variations in the composition, diversity, and functions of bacterial community across the *P. americanum* root-associated compartments, and (3) to examine which environmental factors or soil biochemical properties are important in shaping the structure and carbon substrates utilization diversity of soil bacterial community. All the results could help us better understanding the importance of the interaction between *P. americanum* roots and the soil microorganism during atrazine-polluted soil phytoremediation.

## Materials and Methods

### Soil Samples

The soil used in this research was collected from a farmland in Harbin, Heilongjiang Province which is located in the black soil region of northeast China. The sampled soil was air dried, passed through a 2-mm sieve and detected to be no atrazine. Total organic carbon, ammonium nitrogen, rapidly available phosphorus, rapidly available potassium and pH which are the basic properties of the soil were 20.04 g kg^-1^, 89.33 mg kg^-1^, 70.15 mg kg^-1^, 501.00 mg kg^-1^ and 6.28, respectively.

### Experimental Design and Compartmented System

**FIGURE 1:**
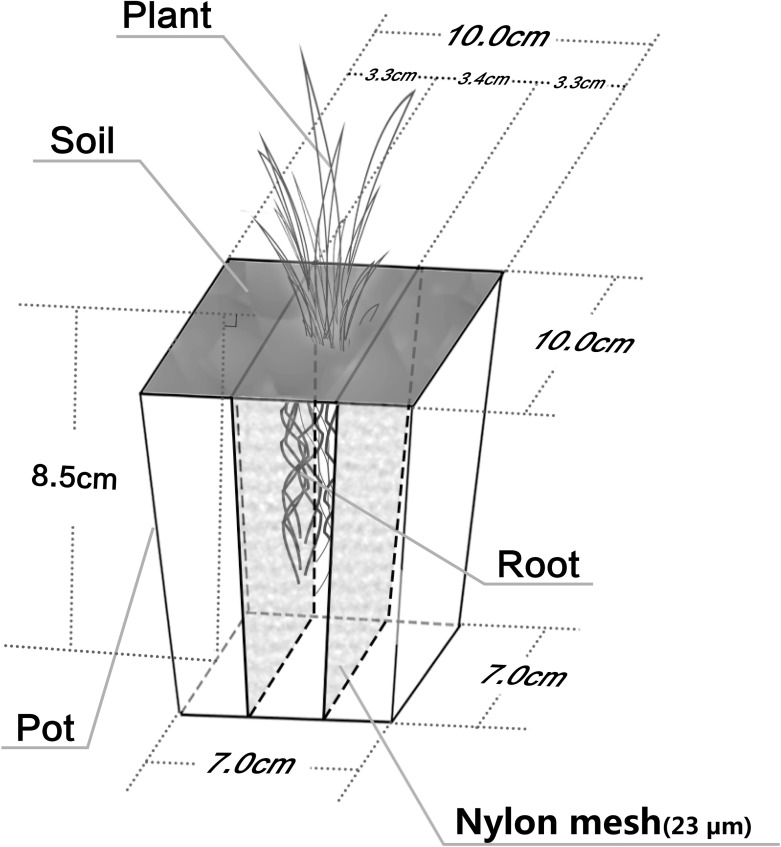
Rhizobox used in phytoremediation treatment (RT). The rhizobox was divided into three parts by a 23 μm nylon mesh. The test plant was only sowed in the middle part and this part represents rhizosphere zone. Additionally, the other two parts in the two side of the rhizobox without sowing test plant were designed as non-rhizosphere.

To illustrate the regulatory role of the *P. americanum* rhizosphere on bacterial functional diversity and the bacterial community structure of atrazine-contaminated soil, three treatments were set up: (1) soil without any addition of atrazine or *P. americanum*, which served as the control treatment (CK); (2) soil without planting of *P. americanum* but with addition of 20 mg kg^-1^ atrazine, which served as pollution treatment (PT); (3) soil with planting of *P. americanum* and addition of 20 mg kg^-1^ atrazine as phytoremediation treatment (RT). To separate the non-rhizosphere soil (RN) from the rhizosphere soil (R) in the phytoremediation treatment (RT), a rhizobox was used (**Figure 1**). A 23 μm nylon mesh was used to divide the rhizobox (100 mm length × 100 mm width × 85 mm height) into three sections: the rhizosphere zone, which was in the center of the rhizobox (34 mm in width), and the two non-rhizosphere zones, which were located on the left and right sides of the rhizobox (each 33 mm in width). Four hundred twenty grams of air-dried soil was put in the rhizobox. In the phytoremediation treatment, *P. americanum* was only sown in the central zone. In addition, plastic pot of the same size containing identical amounts of soil were used in the CK and PT treatments, but without dividing these boxes into three parts and without sowing the seeds.

To prepare the soil used for the treatments of PT and RT, atrazine was dissolved in acetone, followed by being completely mixed with a small part of the soil, and then the spiked soil was put in the fume hood to make acetone vaporize thoroughly. Finally, the soil was mixed into a large amount of the soil homogeneously. The final atrazine concentration in the soil was 20 mg kg^-1^ (dry weight).

Seeds of *P. americanum* were soaked in distilled water for 5 h and then were surface-sterilized in 30% H_2_O_2_ solution for 10 min. Afterward, the sterilized seeds were rinsed several times with deionized water and were placed in a culture dish with moist filter paper for germination overnight at 28°C. The germinated seeds were sown in the rhizosphere zone of the rhizobox mentioned above. Pot experiments were performed in a greenhouse. The temperature of the greenhouse was kept at 27 ± 1°C during the day and 20 ± 1°C during the night. All the samples were watered with distilled water every 2 days to keep the plants at approximately 50% of the water holding capacity. Three replicates were conducted for each treatment mentioned above.

The day of sowing the tested plant seeds described in Section “Experimental Design and Compartmented System” was set as day 0. The soil of CK and PT was sampled on day 0 and the collected samples were named CK0 and PT0, respectively. Additionally, the soil of CK, PT, RN and R were sampled when a 28-day cultural period (as described above) was finished and the collected samples were marked as CK28, PT28, RN28 and R28, respectively. Each sample mentioned above was divided into two sets. One was stored at -20°C until soil microbial community structure and function assessment, and the other was stored at 4°C to measure atrazine concentration and other soil characteristics, such as pH, Eh, catalase activity and urease activity, water-soluble organic carbon and microbial biomass carbon.

### Soil Physicochemical Properties and Microbial Biomass Carbon Determination

Some typical physicochemical properties and the microbial biomass carbon of the soil samples mentioned above were detected by the methods described below. (1) Soil pH was determined in water (1:2.5, soil/water) with a pH meter (Rex PHS-3C, China). (2) Soil Eh was measured with an ORP electrode (Rex 501, China). (3) The water- soluble organic carbon (WSOC) was measured using a TOC analyzer (Shimadzu TOC-VCPN, Japan) according to the method reported by Nan et al. (2016). Soil samples (6 g) were shaken with distilled water (ratio of 1:5, w/v) for 1 h at 25°C and 180 r min^-1^ and centrifuged (4500 rpm for 5 min), and the supernatants were filtered through 0.45 μm filter membrane. The extract was analyzed. (4) Soil catalase activity was determined by measuring the hydrogen peroxide (H_2_O_2_)-catalyzing ability when soil was incubated in H_2_O_2_ solution. Two grams fresh soil was added to 40 mL hydrogen peroxide solution (0.03%, w/v) and cultured at 37°C, 150 r min^-1^ for 30 min. The enzymatic reaction was stopped by adding 5 mL of 3.0 M H_2_SO_4_. Then, 25 mL filtrate was titrated by 0.1 M KMnO_4_ and the soil catalase activity was calculated basing on the change in H_2_O_2_ concentration as reported by Cao et al. (2015). (5) Soil urease activity was determined by a sodium phenolate and sodium hypochlorite spectrophotometry. Five grams of soil (wet weight) was placed in tested tube and 1 mL toluene, 10 mL urea (10%, w/v) and 20 mL citrate buffer (pH = 6.7). The mixture was cultured at 37°C for 24 h, then the solution was filtered and measured by spectrophotometer (Shimadzu UV-1800, Japan) at the wavelength of 578 nm (Cao et al., 2015). (6) The soil microbial biomass carbon (MBC) was measured by the chloroform-fumigation-extraction method (Vance et al., 1987). The extracted organic C was determined using the TOC analyze and a K*_EC_* of 0.45 was used to convert the difference between the organic C extracted with 0.5 M K_2_SO_4_ from the chloroform fumigated and unfumigated soil samples.

### Soil Microbial Physiological Metabolic Characteristics Analysis

Biolog EcoPlates (MicroPlate., BIOLOG Inc., Hayward, CA, United States) were employed to study the microbial physiological metabolic characteristics. Four grams of soil (wet weight) was added to 36 mL of sterilized 0.85% NaCl/water solution. Tenfold serial dilutions were made and the 10^-3^ dilution was added into the Biolog EcoPlates. Then, the plates were cultured at 25 ± 1°C in the incubator in the dark avoided light for 7 days. Color development in the plates was recorded with an automated microplate reader (Biotek Epoch, United States) every 24 h at 590 nm. Plate readings at 96 h of incubation were used to calculate the average well color development (AWCD), Shannon index (H′), Simpson index(D) and McIntosh index(U), since 96 corresponded to the time of maximal microbial growth that allowed the best resolution among the treatments.

### DNA Extraction, PCR and High-Throughput Sequencing

DNA was extracted from the soil samples (0.4 g wet weight) with E.Z.N.A Soil DNA (OMEGA, United States) according to the manufacturer’s instructions. The V4 hypervariable region of bacterial 16S rRNA gene fragments were amplified in triplicate from each of the resulting DNA extracts using the primers 515F (5′-GTG CCAGCMGCCGCGGTAA-3′) and 806R (5′-GGACTACHVGGGTWTCTAAT-3′). The amplification was carried out in 20 μL mixture 4 μL of 5 × FastPfu Buffer, 2 μL of 2.5 mM dNTPs, 0.8 μL of each primer (5 μM), 0.4 μL of FastPfu Polymerase and 10 ng of template DNA. The amplification conditions involved an initial denaturing step at 95°C for 2 min followed by 25 cycles (95°C for 30 s, 56°C for 30 s, 72°C for 30 s) and a final extension at 72°C for 5 min.

Amplicons were purified using QIAquick PCR Purification Kit (Qiagen, China) and quantified using QuantiFluor-ST fluorometer (Promega, United States) according to the manufacturer’s instructions. Then the qualified libraries mentioned above were sequenced pair-end on the Illumina HiSeq System (Illumina, United States) by the sequencing strategy PE250.

### Processing of Sequencing Date

The raw data were quality-filtered using QIIME (version 1.17) with the following criteria: (1) Sequence reads not having an average quality of 20 over a 25 bp sliding window based on the phred algorithm were truncated. Meanwhile, we trimmed and removed the reads with lengths less than 75% of their original length. (2) We removed reads contaminated by adapters (default parameter of 15 base overlapped by reads and adapter, as well as a maximal of 3 bases mismatch allowed). (3) We removed of reads with ambiguous base; (4) removal of low complexity reads that contain more than 10 of the same base consecutively. The filtered paired-end reads were combined to tags based on overlaps by FLASH (v1.2.11). The tags with 97% pairwise identity were binned into operational taxonomic units (OTU) by USEARCH (v7.0.1090). The abundance of each OTU was calculated according to the USEARCH_global method. The most abundant sequence of each OTU was selected as the representative OTU sequence. Taxonomic designation of OTUs was assigned by comparing the representative OTU sequence against the Greengenes database using RDP Classifier (v.2.2). The bacterial community structures diversity of the samples were further analyzed according to the OTU taxonomic richness and number.

### Statistical Analysis

The results of the soil typical physicochemical property for each experiment treatment were given as means and standard deviations of three replicates. Statistical significance between treatments was performed using SPSS 19.0 with two-way ANOVA and least significant difference (LSD) at *p* < 0.05.

Principal component analysis (PCA) was performed in Canoco for Windows 4.5 to compare the differences of the microbial physiological metabolic characteristics of the studied treatments based on Biolog EcoPlates data. Redundancy analysis (RDA) was carried out in Canoco for Windows 4.5 to determine which soil environmental variables best explained the changes in the frequency distributions of microbial metabolic functions under various treatments.

The bacterial community structure diversity indices were calculated using Mothur (v1.31.2). Principal coordinate analyses based on pairwise unweighted and weighted UniFrac distances were calculated in the “ade4” package of R software (v3.1.1). The information of common and unique OTUs among various treatments was plotted by “VennDiagram” package of R (v3.1.1). The log_10_-transformed relative abundance of genus-level OTUs was used to construct a heat map using the “gplots” package for R software (v3.1.1). A hierarchical cluster analysis was performed using BrayeCurtis distances. The relationship between the bacterial community structure and environmental factors was visualized according to redundancy analysis (RDA), which was performed with Canoco for Windows 4.5.

## Results

### Soil Property and Microbial Biomass Carbon (MBC)

The typical physical and biochemical characteristics, such as pH, Eh, water-soluble organic carbon (WSOC), catalase and urease activity, and MBC of the soil samples collected during the experimental period are summarized in **Table 1**. CK0 and PT0 only differed in catalase activity (0.56 mg KMnO_4_ g^-1^ h^-1^ and 0.71 mg KMnO_4_ g^-1^ h^-1^, respectively). However, CK, PT, R and RN exhibited various soil physicochemical properties and MBC contents on day 28. Nearly all the indices mentioned above (except Eh and MBC) of R28 were significantly higher than those of CK28 and PT28 (*P* < 0.05). In addition, R28 presented higher pH, catalase activity, urease activity and WSOC than RN28, whereas Eh and MBC were significantly lower in R28 than RN28. Furthermore, CK28 presented higher catalase activity and lower Eh than CK0. On the other hand, PT28 and PT0 only differed in Eh.

**Table 1:** Soil physicochemical properties and microbial biomass carbon.

	Day 0		Day 28			
		
	CK0	PT0	CK28	PT28	RN28	R28
pH	6.28 ± 0.06a	6.35 ± 0.10a	6.47 ± 0.01C	6.72 ± 0.03B	6.82 ± 0.03B	7.59 ± 0.11A
Eh(mV)	407 ± 12a	402 ± 7a	371 ± 2B	379 ± 7B	400 ± 2A	373 ± 4B
CAT(mg KMnO_4_ g^-1^ h ^-1^)	0.56 ± 0.01b	0.74 ± 0.03a	0.76 ± 0.07C	0.80 ± 0.09BC	0.89 ± 0.03B	1.16 ± 0.02A
URE(mg NH_4_-N g^-1^)	24.06 ± 1.43a	24.73 ± 1.88a	23.63 ± 0.42C	24.31 ± 1.51C	30.29 ± 0.67B	38.81 ± 1.09A
WSOC(mg kg^-1^)	357.75 ± 48.75a	364.17 ± 78.46a	408.69 ± 27.35B	344.65 ± 30.56C	451.06 ± 18.83B	523.60 ± 24.16A
MBC(mg kg^-1^)	269.21 ± 68.87a	232.69 ± 83.06a	254.14 ± 1.94C	331.60 ± 81.37BC	452.06 ± 37.19A	364.31 ± 40.56AB


*CAT, catalase; URE, urease; WSOC, water-soluble organic carbon; MBC, microbial biomass carbon; CK, control treatment; PT, pollution treatment (initial atrazine concentration was 20 mg kg^-*1*^); RN, non-rhizosphere zone of the phytoremediation treatment; R, rhizosphere zone of phytoremediation treatment. The sampling points were day 0 and day 28, and the day of sowing the plant seeds was set as day 0. The results are given as the means ± SD (*n* = 3). Different lower-case letters indicate significantly different values between samples of CK and PT at day 0 (*p* < 0.05). Different capital letters indicate significantly different values between samples of CK, PT, RN, and R at day 28 (*p* < 0.05).*

### Soil Microbial Function Assessment

The results of sampled soil microbial function assessment are shown in **Table 2**. The soil microbial functional diversity indices, such as AWCD, H′, D and U, were not significantly different between CK0 and PT0. In contrast, the soil on day 28 in the four treatment groups exhibited various microbial function diversities. Nearly all the indices mentioned above (except D) of R28 were significantly higher than those of CK28 and PT28 (*P* < 0.05). In addition, R28 presented higher AWCD, H′, and U than did RN28, whereas D was not significantly different between R28 and RN28. Furthermore, CK28 presented lower AWCD, H′, D and U than did CK0. On the other hand, PT28 presented lower AWCD and U than PT0 did.

**Table 2:** Soil bacterial community functional diversity indices of all samples.

Treatments		AWCD	H′	D	U
0 day	CK0	0.94 ± 0.06a	3.02 ± 0.08a	0.94 ± 0.00a	6.91 ± 0.39a
	PT0	0.74 ± 0.16a	2.98 ± 0.05a	0.94 ± 0.00a	5.51 ± 0.95a
28 days	CK28	0.13 ± 0.03C	1.83 ± 0.22C	0.74 ± 0.06B	2.08 ± 0.37C
	PT28	0.14 ± 0.06C	2.59 ± 0.42B	0.88 ± 0.07A	1.40 ± 0.35C
	R28	0.96 ± 0.15A	3.17 ± 0.04A	0.95 ± 0.00A	6.43 ± 0.86A
	RN28	0.51 ± 0.03B	2.95 ± 0.06AB	0.94 ± 0.00A	3.98 ± 0.27B


*AWCD, average well color development; H′, Shannon index; D, Simpson index; U, McIntosh index; CK, control treatment; PT, pollution treatment (initial atrazine concentration was 20 mg kg^-*1*^); RN, non-rhizosphere zone of the phytoremediation treatment; R, rhizosphere zone of phytoremediation treatment. The sampling points were day 0 and day 28, and the day of sowing the plant seeds was set as day 0. The results are given as the means ± SD (*n* = 3). Different lower-case letters indicate significantly different values between samples of CK and PT at day 0 (*p* < 0.05). Different capital letters indicate significantly different values between samples of CK, PT, RN, and R at day 28 (*p* < 0.05).*

**FIGURE 2:**
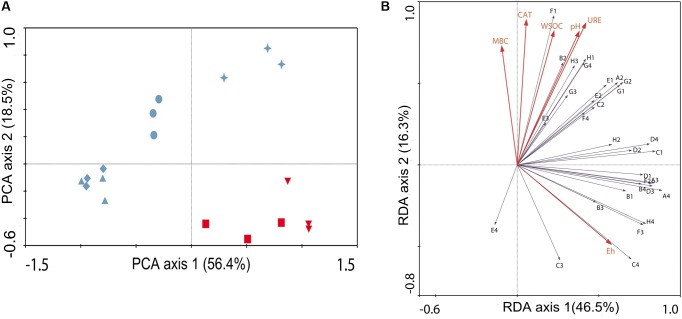
Analysis of functional diversity of bacterial communities in each researched treatment. **(A)** principle component analysis performed with the absorbance values of 31 carbon sources in Biolog ECO plates, measured at 96 h of incubation after adding serial dilutions of soil collected from the treatments. Red downturned triangle (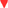) represents CK0, red square (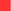) represents PT0, blue upturned triangle (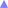) represents CK28, blue diamond (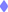) represents PT28, blue circle (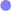) represents RN28 and blue star (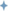) represents R28. **(B)** RDA was performed with absorbance values mentioned above and the significant environmental parameters (red arrows, explanatory variables), such as pH, catalase activity (CAT), urease activity (URE), Eh, water soluble organic carbon (WSOC) and microbial biomass carbon (MBC). The arrows show the positions of functional microbial groups (metabolizing specific substrates): A2, β-Methyl-D-glucoside; A3, D-Galactonic acid γ-Lactone; A4, L-Arginine; B1, Pyruvic acid methyl ester; B2, D-Xylose; B3, D-Galacturonic acid; B4, L-Asparagine; C1, Tween 40; C2, I-Erythritol; C3, 2-Hydroxy benzoic acid; C4, L-Phenylalanine; D1, Tween 80; D2, D-Mannitol; D3, 4-Hydroxy benzoic acid; D4, L-Serine; E1, α-Cyclodextrin; E2, *N*-Acetyl-D-glucosamine; E3, γ-Hydroxybutyric acid; E4, L-Threonine; F1, Glycogen; F2, D-Glucosaminic acid; F3, Itaconic acid; F4, Glycyl-L-glutamic acid; G1, D-Cellobiose; G2, Glucose-1-phosphate; G3, α-Ketobutyric acid; G4, Phenylethylamine; H1, α-D-Lactose; H2, D,L-α-Glycerol phosphate; H3, D-Malic acid; and H4, Putrescine.

PCA was performed to reduce the dimensionality of the Biolog EcoPlate data set, as well as to compare the differences in the microbial physiological metabolic characteristics of the researched treatments. The substrate utilization patterns of the researched treatments are shown in **Figure 2A**. The PCA of the substrate utilization patterns extracted two principle components, which explained 74.9% of the total variance together. In addition, the first principle component (PC1) exhibited great power of separation, as it explained 56.4% of the total variance. The PC1 axis showed that the carbon substrate utilization pattern of CK28 was significantly different from that of CK0. Similarly, PT28 exhibited a different carbon substrate utilization pattern from that of PT0. Moreover, PT28 and CK28 were located together, and RN28 was separated from PT28, by PC2. In addition, R28 was completely separated from RN28 and PT28.

**Figure 2B** shows how the soil microbial community functional diversity varied with the potential explanatory variables. Four RDA axes were extracted, and the eigenvalues for these axes were 0.465, 0.163, 0.054, and 0.022, respectively. In addition, the variance in soil microbial functional data could be better explained by first RDA axis, while the soil microbial functional data exhibited a positive correlation with environmental data, with the correlation coefficient of 0.909. The results of the RDA also suggest that functional microbial groups among various treatments were significantly affected by the studied environmental variables, such as Eh, MBC, catalase activity and urease activity. These environmental variables, respectively, explained 12.3, 8, 5.9, and 4.5% of the total variance in the soil microbial functional data. In addition, the Eh of soil positively correlated with the use of L- phenylalanine (C4). Urease activity was strongly negatively correlated with the use of L-threonine (E4) and strongly positively correlated with the use of D-xylose (B2). Moreover, the use of the carbon substrates, such as D-malic acid (H3), α-D-lactose (H1) and phenylethylamine (G4), exhibited higher responses to urease activity. Furthermore, other carbon substrates, including L-threonine (E4) and 2-hydroxy benzoic acid (C3), exhibited a lower response in the proximity of the MBC and catalase activity.

### Soil Bacterial Community Diversity

**FIGURE 3:**
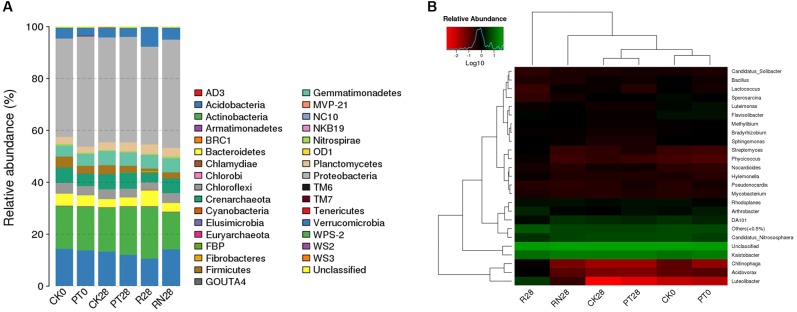
Comparison of the bacterial community structures of the researched treatments at **(A)** phylum level and **(B)** genus level. **(A)** showed the relative abundance of different bacterial phyla by bar plot and **(B)** exhibit the species of clustering heat map based on the relative abundance of different bacterial genus within the researched treatments, including CK, control treatment; PT, pollution treatment; RN, non-rhizosphere zone of the phytoremediation treatment; R, rhizosphere zone of phytoremediation treatment. The sampling point was day 0 (CK0 and PT0) and day 28 (CK28, PT28, R28 and RN28) respectively.

A total of 167,992 high-quality 16S rRNA gene tags generated from all samples were clustered into 2686 OTUs. The relative abundances of the OTUs mentioned above at the phylum level are illustrated in **Figure 3A**. In total, 33 identified phyla were observed. *Proteobacteria*, *Actinobacteria*, and *Acidobacteria* were the three dominant phyla in all soil samples. The relative abundance of *Proteobacteria* phyla was significantly higher in PT0 (42.50%) than CK0 (37.70%). Furthermore, the relative abundances of *Actinobacteria* (19.90%), *Verrucomicrobia* (7.54%), *Bacteroidetes* (6.18%), and *Cyanobacteria* (0.33%) in R28 were significantly higher than those of other treatments. In contrast, the relative abundances of *Proteobacteria* and *Acidobacteria* were lower than other treatments. Moreover, *Fibrobacteres* phylum was only found in R28. In addition, the genera *Kaistobacter*, *Candidatus_Nitrososphaera* and *Arthrobacter* were the dominant genera of the present samples. Particularly, the relative abundances of *Luteolibacter*, *Streptomyces Phycicoccus*, and *Chitinophaga* in R28 were significantly higher than those in other samples, while there were lower relative abundances of *Sporosarcina*, *Lactococcus*, and *Kaistobacter* in R28 than other samples (**Figure 3B**).

The alpha bacterial community diversity indices, such as observed species, including Chao, ACE, Shannon-Weaver and Simpson, are shown in **Table 3**. PT0 exhibited lower richness and diversity compared to the CK0 according to the four calculated indices in **Table 3**. Furthermore, significantly greater diversity was observed in R28 compared to CK28, PT28 and RN28 based on their Shannon indices, while the difference in bacterial richness and diversity among CK28, PT28 and RN28 was not obvious. The time period changed the bacterial richness of CK treatments slightly, as the Chao of CK0 and CK28 were 1800.860 and 1790.369, respectively, and the ACE indices were 1860.425 and 1851.134.

**Table 3:** Bacterial community richness and diversity indices.

Treatments		Chao	ACE	Shannon	Simpson
0 day	CK0	1800.860	1860.425	5.804	0.015
	PT0	1666.950	1722.172	5.601	0.020
28 days	CK28	1790.369	1851.134	5.685	0.018
	PT28	1749.336	1807.963	5.732	0.018
	R28	1736.781	1796.465	5.821	0.013
	RN28	1754.709	1818.818	5.670	0.019


*CK, control treatment; PT, pollution treatment (initial atrazine concentration was 20 mg kg^-*1*^); RN, non-rhizosphere zone of the phytoremediation treatment; R, rhizosphere zone of phytoremediation treatment. The sampling points were day 0 and day 28, and the day of sowing the plant seeds was set as day 0.*

**FIGURE 4:**
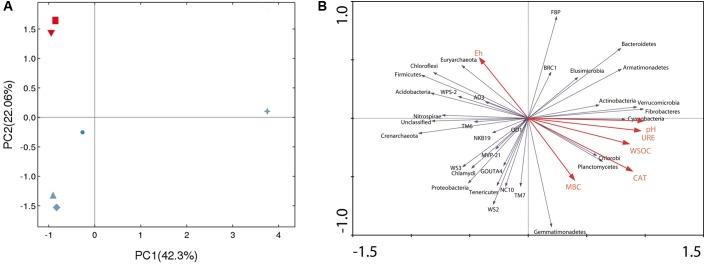
**(A)** Principle component analysis of soil bacterial community composition based on operational taxonomic units abundance of the different treatments, CK, control treatment; PT, pollution treatment; RN, non-rhizosphere zone of the phytoremediation treatment; R, rhizosphere zone of phytoremediation treatment. The sampling point was day 0 and day 28 respectively. Red downturned triangle (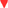) represents CK0, red square (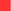) represents PT0, blue upturned triangle (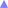) represents CK28, blue diamond (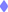) represents PT28, blue circle (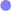) represents RN28 and blue star (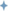) represents R28. **(B)** Ordination plots of the results from the redundancy analysis to identify the relationships among the bacterial populations (blue arrows), soil physico-chemical characteristics and microbial biomass carbon (red arrows). CAT, catalase activity; URE, urease activity; WSOC, water soluble organic carbon; MBC, microbial biomass carbon (MBC).

PCA was applied to identify the differences in bacterial community structure within all the researched treatments. The two principle components (PC1 and PC2) in **Figure 4A** explained 64.36% of the total variance. In addition, the six treatments of this study clustered into four groups. R28 was widely separated from the other five treatments. However, CK28 and PT28, as well as CK0 and PT0, were grouped together and clustered into two respective groups. RN28 itself was clustered into a new group, but it was located near CK28 and PT28.

RDA analysis was performed to show the effect of main soil physicochemical and biological characteristics on the bacterial communities (phylum level). **Figure 4B** shows that the first two axes explained 86.5% of the total variance, indicating that pH, urease activity, catalase activity, and WSOC were the most influential factors driving the changes in the composition and diversity of the bacterial communities. Specifically, the soil pH was strongly negatively correlated with *Nitrospirae* and *Crenarchaeota*, while it was strongly positively correlated with *Cyanobacteria*, *Fibrobacteres* and *Verrucomicrobia*. Urease activity was strongly negatively correlated with *Acidobacteria*. Catalase activity was strongly negatively correlated with *Chloroflexi* and *Firmicutes* but strongly positively correlated with *Chlorobi* and *Planctomycetes. Acidobacteria* and *Firmicutes* exhibited higher responses to WSOC.

## Discussion

The plants of the *Pennisetum* genus exhibits tolerance to herbicide atrazine and potential to decrease the atrazine residual level in the rhizosphere (Zhang et al., 2014; Jiang et al., 2016). A higher microbial biomass might be the main reason for the enhanced degradation of atrazine in the rhizosphere (Singh et al., 2004). Therefore, the rhizosphere is widely considered as a hot spot of pollutants rhizoremediation for its higher microbial activity (Velasco et al., 2013). Furthermore, it has been proved that the high biomass and diversity microbita in rhizosphere is mainly due to the interaction of plant and microorganism during the rhizoremediation period (Velasco et al., 2010; Velasco et al., 2013). However, there is little detailed information about the differences in the soil physicochemical properties, microbial metabolize and bacterial community diversitycharacteristics between the rhizosphere and non-rhizosphere soil of *Pennisetum* genus plants in remediation of contaminated soil. This paper is mainly intends to further illustrate the relationships among *P. americanum*, rhizosphere soil physicochemical properties and bacterial community traits during the phytoremediation of atrazine-contaminated soil.

Catalase in soil is responsible for removal of the hydrogen peroxide (H_2_O_2_) and alleviating the oxidative damage to microorganisms and plants. **Table 1** shows that significantly greater catalase activity was found in PT0 than CK0. This phenomenon might be mainly because of the oxidative stress response of soil microorganisms to the addition of atrazine. This inference could be further supported by the well-known viewpoint that atrazine causes oxidative stress on various types of organisms (Zhang et al., 2012; Jiang et al., 2016). Catalase activity could also be used to evaluate the metabolic activity of soil microbial communities (Samuel et al., 2011), and urease activity exhibits a strong correlation with the organic nitrogen transfer ability by microbes. Therefore, the higher catalase and urease activities in R28 suggest that the *P. americanum* rhizosphere could accelerate the metabolic activity of soil microorganisms by releasing various types of nutrient substances (root exudates) to boost the functional microbial survival, or by changing the soil micro-environment to make it favorable to the microorganisms mentioned above. These possibilities are in line with the result that the concentration of water-soluble organic carbon (WSOC), which might be released by the roots of *P. americanum*, in R28 was higher than those of other treatments. They also could be supported by the result that the pH in the rhizosphere of *P. americanum* was much closer to the suitable pH range (6–8) for microorganisms.

The effect of the *P. americanum* rhizosphere on the bacterial catabolic ability was assessed by determining the community-level physiological profiles (CLPP) of soil bacteria using Biolog EcoPlates. Meanwhile, redundancy analysis (RDA) was employed to further investigate the interaction of environmental factors and the carbon-containing substrate utilization characteristics of the soil bacteria (**Figure 3B**). We found that the studied soil physical, chemical and biological properties, such as Eh, MBC, catalase activity and urease activity, exhibited strong positive or negative correlations with the utilization of some types of substrates in the Biolog EcoPlates. Combined with the results described above that the soil physical, chemical and biological properties were affected by the *P. americanum* rhizosphere, it is reasonable to infer that the bacterial catabolic ability of the *P. americanum* rhizosphere might be different from those of other treatments. CLPP-based principal component analysis (PCA) suggested that the substrate utilization pattern of R28 was completely different from other treatments, based on the two principal components in **Figure 3A**. Moreover, the higher bacterial functional diversity indices (AWCD, H′, and U) in R28 revealed that the soil in *P. americanum* rhizosphere exhibited much greater carbon substrate utilization ability, since the indices mentioned above are commonly proposed to measure the functional diversity or catabolic ability of bacterial community (Villeger et al., 2008). These results might partly illustrate why higher organic pollutants removal efficiency could be found in rhizosphere soil (Li et al., 2011; Blaud et al., 2015).

It has been thought that the soil microbial activity especially the carbon utilization ability could be affected by the species-specific root exudates released from various types of plant species, since the root exudates are the most important sources of readily available carbon for rhizosphere microorganisms (Martineau et al., 2014; Yuan et al., 2016). Because the microorganisms differ in their ability to metabolize and compete for different carbon sources, it is reasonable to consider that the structure of microorganism communities might change during the variation of microorganism functional diversity, as well as the soil physical and chemical properties (Berg and Smalla, 2009). In this paper, high throughput sequencing technology based on the Illumina HiSeq platform was selected to access the bacterial community information of the researched treatments. The redundancy analysis (RDA) based on the bacterial community information and the soil physical and chemical properties further showed that the detected soil properties, such as pH, urease activity, catalase activity, and WSOC, affected the bacterial composition of soil samples collected from various treatments. Since the typical soil physical and chemical properties of the *P. americanum* rhizosphere were different from that of non-rhizosphere treatments (**Table 1**), it also can be concluded that the test plant *P. americanum* could shape the rhizosphere environment, as well as the bacterial community. Additionally, the pincipal component analysis (PCA) of the bacterial community information extracted from the treatments in **Figure 4A** further show that the *P. americanum* rhizosphere exhibited a different bacterial community characteristics. Therefore, a strong evolutionary relationship between *P. americanum* and bacteria might exist in the rhizosphere. These observations are in agreement with results by that Lacalle et al. (2018) that *Brassica napus* plants not only increased the activity of microbial communities in contaminated soils, but also its functional diversity by creating suitable conditions for microbial growth in the rhizosphere.

**FIGURE 5:**
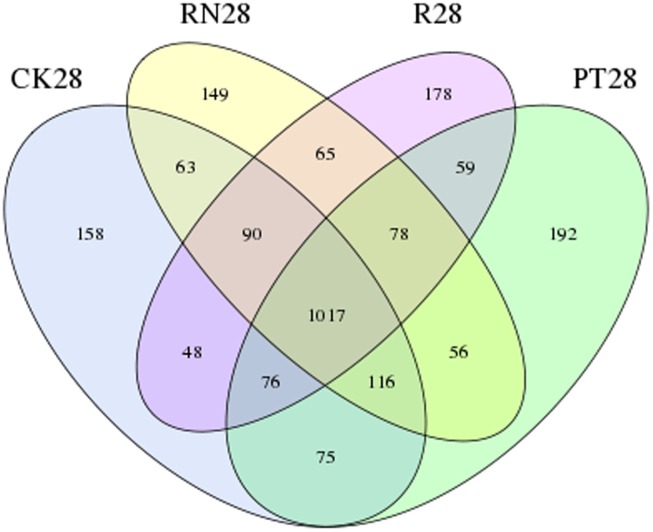
Venn diagram of operational taxonomic units (OTUs) distribution among the samples collected at day 28 of the researched treatments, including CK, control treatment; PT, pollution treatment; RN, non-rhizosphere zone of the phytoremediation treatment; R, rhizosphere zone of phytoremediation treatment. The number in the Venn diagram depicting the shared and unique OTUs present in the four treatments mentioned above.

This study also found that though R28 exhibit a higher bacterial diversity (Shannon indice was 5.821), the greatest bacterial species richness (Chao and ACE) was not found in R28 (**Table 3**). This phenomenon might have been due to the succession of the bacterial communities in the *P. americanum* rhizosphere which triggers an environmental filtering shift of bacteria community composition (Zappelini et al., 2015; Chen et al., 2016). This inference is in line with the data presented in this study that some genera of bacteria, such as such as *Arthrobacter*, *Chitinophaga*, *Streptomyces*, *Sporosarcina* and *Phycicoccus*, were very sensitive to the *P. americanum* rhizosphere environment, as the relative abundances of these genera in R28 were significantly different from those of other treatments. In addition, we found that there were 178 unique OTUs in R28 by comparing the bacterial community composition with those of CK28, PT28 and RN28 (**Figure 5**). Taxonomic analysis indicated that some of the unique OTUs mentioned above belonged to the phyla *Chloroflexi* and *Cyanobacteria*, the order *Acidimicrobiales*, as well as the genera *Paenibacillus* and *Rhizobium*, which can facilitate soil nutrient cycling (Bustamante et al., 2006; Rodrigues et al., 2015; Zimmermann et al., 2015; Redding et al., 2016). In addition, some other OTUs which represent the bacteria with the potential to degrade organic pollutants were found in the *P. americanum* rhizosphere. These OTUs consisted of the genus *Sphingobium* which could participate in the degradation of herbicide (Sun et al., 2008), the genus *Mycoplana* which can decompose 2,4-dichlorophenol (Manikandan et al., 2008), and the family *Sphingomonadaceae*, which degrades aromatic compounds (Lafortune et al., 2009). It is worth to note that the class *Fibrobacteria* and the order *Rhizobiales*, two types of bacteria frequently appearing around the rhizosphere that can decompose fiber and fix nitrogen, respectively, only were detected in R28. These results further suggest that the *P. americanum* also could effectively boost the potential of nutrient metabolism and pollutants degradation in rhizosphere by enhancing the kind or abundance of the bacteria with the corresponding ecology function. It could be greatly supported by the results described above that the abundance of the *Arthrobacter* genus bacteria was obviously enhanced in rhizosphere of *P. americanum* (**Figure 2B**), since much more atrazine-degrading strains has been identified as *Arthrobacter* genus (Zhang et al., 2011). This inference could be further supported by our previous published results that *P. americanum* could obviously accelerate the atrazine removal in soil than that of the treatment without any plant (Zhang et al., 2014). Indeed, further research might continually focus on the variety of microbial functional genes, which responsible for atrazine degradation and soil nutrient cycling, to further illustrate the phytoremediation mechanism of atrazine-contaminated soil by *P. americanum*.

## Conclusion

*Pennisetum americanum* planted in atrazine-contaminated soil shaped the bacterial communities and enhanced the bacterial functional diversity of the rhizosphere by re-shaping the soil physicochemical properties, such as catalase activity, urease activity, WSOC and pH, to be more suitable to soil microorganisms. Additionally, some unique types of bacteria that could facilitate soil nutrient cycling or organic pollutant degradation were only found in the rhizosphere of *P. americanum*. This study provides insight into how that the interaction between the *P. americanum*, soil physicochemical environment as well as the soil bacterial properties (community and functional diversity) plays an important role during the phytoremediation process of atrazine-contaminated soil.

## Author Contributions

BC, as the first author of this manuscript, was mainly responsible for writing the whole manuscript and analyzing the results about the microorganism community diversity. YZ and ZJ designed the whole experiment together and calculated the data. ML mainly prepared the rhizobox that was used in this research, as well as did much work for soil microbial physiological metabolic characteristics analysis using Biology Eco plates. FY worked on the soil sample collection. ZW detected the soil physicochemical properties of the soil samples. DJ was responsible for the extraction of the soil microorganism DNA and the detection of the soil microbial biomass carbon. In addition, ZJ was also responsible for submitting the manuscript to the journal.

## Conflict of Interest Statement

The authors declare that the research was conducted in the absence of any commercial or financial relationships that could be construed as a potential conflict of interest.
